# Hepatectomy leads to loss of TRAIL-expressing liver NK cells via downregulation of the CXCL9-CXCR3 axis in mice

**DOI:** 10.1371/journal.pone.0186997

**Published:** 2017-10-31

**Authors:** Takuya Yano, Masahiro Ohira, Ryosuke Nakano, Yuka Tanaka, Hideki Ohdan

**Affiliations:** Department of Gastroenterological and Transplant Surgery, Applied Life Sciences, Institute of Biomedical & Health Sciences, Hiroshima University, Hiroshima, Japan; University of Navarra School of Medicine and Center for Applied Medical Research (CIMA), SPAIN

## Abstract

Liver-resident natural killer (NK) cells express TNF-related apoptosis-inducing ligand (TRAIL), a critical molecule for NK cell-mediated tumor cell killing. We previously reported that TRAIL expression in liver NK cells decreases markedly after hepatectomy; however, the mechanism underlying this drastic alteration remains unknown. In this study, we assessed the role of chemokine signaling in liver-resident NK cells during the perioperative period of hepatectomy. The expression levels of various chemokine receptors on liver-resident NK cells and their associations with TRAIL expression were analyzed by flow cytometry. The expression of various intrahepatic chemokines/cytokines was analyzed after 70% hepatectomy in mice by quantitative RT-PCR and flow cytometry. We further investigated whether polyinosinic—polycytidylic acid (poly I:C)-induced NK cell activation could ameliorate TRAIL expression in the liver after 70% hepatectomy in *CXCR3*^*-/-*^ and wild-type mice. TRAIL^+^ NK cells strongly and exclusively expressed CXCR3, and the expression of its ligand CXCL9 was significantly decreased in the liver after hepatectomy. The kinetics of hepatic CXCL9 expression resembled the changes in hepatic TRAIL^+^ NK cells after hepatectomy. Among liver-resident mononuclear cells, CXCL9 was predominantly secreted by macrophages in response to interferon-γ stimulation. Although the administration of poly I:C, an inducer of interferon-γ, increased hepatic CXCL9 levels in both *CXCR3*^*-/-*^ and wild-type mice even after hepatectomy, only wild-type mice exhibited the recovery of TRAIL expression on NK cells. Partial hepatectomy remarkably reduced the proportion of TRAIL-expressing NK cells in the liver via the downregulation of the CXCL9–CXCR3 axis in mice. These findings extend our knowledge of the factors contributing to hepatocellular carcinoma recurrence after hepatectomy.

## Introduction

Natural killer (NK) cells are an important defense mechanism against invading infectious microbes and neoplastic cells, as they exert an effector function that is not dependent on priming [1, 2]. They are abundant in mouse livers, but not in peripheral lymphatics [3, 4]. NK cell abundance also differs between liver and peripheral blood in humans, but the mechanism underlying this anatomically biased distribution is unclear. Tumor cell cytotoxicity is higher for liver NK cells than spleen or peripheral blood NK cells in both rodents and humans [3–5]. NK cells exhibit reduced anti-tumor activity after partial hepatectomy; therefore, immunocompromised patients after partial hepatectomy or partial liver transplantation are susceptible to hepatocellular carcinoma recurrence [6–8].

Various mechanisms are involved in the control of neoplastic cells by NK cells. For example, cytolytic granules that contain perforin, granzymes, and granulysin are directly released via the granule exocytosis pathway [9, 10]. Another mechanism is mediated by death-inducing ligands, such as Fas ligand and TNF-related apoptosis-inducing ligand (TRAIL) [11–13]. TRAIL, an Apo2 ligand, is a type II transmembrane protein that belongs to the TNF family. There are two types of TRAIL receptors, i.e., one that can induce apoptotic signals and another that acts as a decoy receptor [14]. The binding of NK cell TRAIL to its apoptotic receptors (death receptors) on target cells mediates target cell lysis and functions via the extrinsic apoptosis pathway (as opposed to the mitochondrial apoptosis pathway) [15].

Liver-resident DX5^−^ NK cells exclusively express TRAIL and induce vigorous cytotoxicity against hepatoma cells in naïve mice [16, 17]. We previously found that partial hepatectomy significantly decreases TRAIL expression on liver NK cells, weakening their immune activity against neoplastic cells, thereby promoting cancer recurrence after hepatectomy [18]. However, the mechanisms underlying this remarkable alteration in TRAIL expression remain unclear.

It has been demonstrated that the transcription factor T-bet determines developmental stability in immature NK cells with constitutive expression of TRAIL. In addition, maturation, in which expression of TRAIL is reduced and that of the Ly49 receptor and integrin DX5 is induced, requires the transcription factor Eomes [19]. Hence, the substantial reduction in the TRAIL^+^ NK cell proportion in the liver after hepatectomy might be explained by NK cell stability during maturation in the liver. Alternatively, liver-resident NK cell chemotaxis might affect NK cell distribution/trafficking, since these cells express different adhesion molecules and chemokine receptors at different developmental stages and can therefore be recruited to different anatomical sites [20]. Furthermore, local microenvironmental conditions can lead to NK cell differentiation, yielding tissue-specific NK cells. In the present study, we assessed the roles of chemokine signaling in liver-resident NK cells during the perioperative period of hepatectomy and investigated the mechanism by which TRAIL^+^ NK cells disappear from the liver after hepatectomy.

## Materials and methods

### Ethics statement

This study was performed in strict accordance with the Guide for the Care and Use of Laboratory Animals and the local committee for animal experiments. The experimental protocol was approved by the Ethics Review Committee for Animal Experimentation of the Graduate School of Biomedical Sciences, Hiroshima University (Permit A13-112). All animal experiments were performed according to the guidelines established by the US National Institutes of Health (1996). This work was carried out, in part, at the Research Facilities for Laboratory Animal Science, Natural Science Center for Basic Research and Development (N-BARD), Hiroshima University.

### Mice

C57BL/6J (B6) (H-2b) mice were purchased from CLEA Japan, Inc. (Osaka, Japan). CXCR3-deficient (B6.129P2-Cxcr3tm1Dgen/J, *CXCR3*^*-/-*^) mice were obtained from the Jackson Laboratory (Bar Harbor, ME, USA). The mice were housed in the animal facility of Hiroshima University, Japan, in a pathogen-free microenvironment. Male and female mice were used between 8 and 12 weeks of age. Animal welfare was carefully ensured by experienced operators every day. Mice were euthanized by cervical dislocation after isoflurane inhalation, when indicated. All efforts were made to minimize the suffering of animals for the duration of their lives and during the sacrifice of mice.

### Partial hepatectomy of mice

Prior to surgery, mice were anesthetized by intraperitoneal injection of xylazine (5 mg/kg body weight) and ketamine (100 mg/kg body weight). Under anesthesia, the large median lobe of the liver and the left lateral lobe were securely ligated and subsequently excised. Portions of the hepatic parenchyma, ranging from 65–75% of the total liver mass, were removed in this manner. After operation, mice were placed on a heating pad for postoperative recovery. When indicated, mice were treated by intraperitoneal injection of polyinosinic—polycytidylic acid (poly I:C; 500 μg/mouse) (Sigma, St. Louis, MO, USA), 48 h before harvesting.

### Isolation of leukocytes

Under anesthesia, liver lymphocytes were prepared as previously described [21]. Briefly, after injection of 1 mL of phosphate-buffered saline (PBS) supplemented with 10% heparin via the portal vein, the liver was excised and perfused with 50 mL of PBS supplemented with 0.1% ethylenediaminetetraacetic acid. From the liver perfusate, blood cells were harvested by centrifugation, and erythrocytes were then removed using ammonium chloride potassium lysing buffer.

### Flow cytometric analysis

All analyses were performed using a FACSCanto II Cytometer (BD Biosciences, Mountain View, CA, USA). To phenotype NK cell surface markers, liver and spleen leukocytes were stained with the following mAbs: anti-NK1.1 (PK136), anti-CD69 (H1.2F3), anti-CXCR3 (CD183), anti-CCR5 (CD195), anti-CCR7 (CD197), and anti-CD19 (1D3) (all obtained from BD Pharmingen, San Diego, CA, USA); anti-TCRβ chain and anti-TRAIL (CD253) (BioLegend, San Diego, CA, USA); anti-CX3CR1 and anti-CXCR6 (R&D Systems, Minneapolis, MN, USA); and anti-TRAIL (CD253) (eBioscience, San Diego, CA, USA). Chemokine production by lymphocytes was measured by staining for a combination of cell surface and cytoplasmic mAb staining and subsequent flow cytometric analysis. Briefly, 4 h after treatment with Protein Transport Inhibitor (containing brefeldin A; BD GolgiPlug, BD Biosciences), lymphocytes were stained with anti-NK1.1-FITC and anti-TCRβ-APC (BD Bioscience) or anti-F4/80-APC (eBioscience), permeabilized with fixation/permeabilization solution, and incubated with anti-CXCL9-PE (MIG-PE; eBioscience).

### Quantitative RT-PCR

Total RNA was extracted from mouse samples, using the RNeasy Mini Kit (Qiagen, Venlo, The Netherlands), and cDNA was synthesized using a QuantiTect Reverse Transcript Kit (Qiagen). PCRs were performed in triplicate in 20-μL reaction volumes using SYBR Green PCR Master Mix (Applied Biosystems, Foster City, CA, USA) according to the manufacturer’s specifications. Gene expression levels were measured with a Rotor-Gene Q (Qiagen, Hilden, Germany). The amplification protocol consisted of denaturation at 95°C for 5 min, followed by 40 cycles of 95°C for 5 s and 60°C for 10 s. Relative quantities were calculated using the ΔΔCt formula and normalized against the transcript levels of the housekeeping gene beta-2 microglobulin. The PCR primers used for the gene analysis are shown in S1 Table.

### Cytometric bead assay

To examine chemokine levels, liver tissues were homogenized in lysis buffer (Cell Lysis Buffer; Cell Signaling Technology, Danvers, MA, USA). Protein samples were centrifuged at 10,000 rpm for 10 min. Supernatants were collected and protein concentrations were determined using the Pierce BCA Protein Assay Kit (Thermo Fisher Scientific). CXCL9 in the liver was analyzed using the cytometric bead array (CBA) with Mouse MIG (CXCL9) Flex Sets (BD Bioscience) according to the manufacturer’s instructions.

### Cell migration assay

Migration of purified NK cells was assessed based on their movement through a 5-μm-pore Transwell insert (Corning Costar Corp., New York, NY, USA). Recombinant CXCL9 (R&D Systems), at 100 ng/mL, was added to the outer well of the Transwell plate in a final volume of 600 μL (RPMI 1640 + 10% FCS). NK cells were purified by magnetic bead separation (Miltenyi Biotec, Bergisch Gladbach, Germany), and 1 × 10^6^ NK cells were then added to the inner well in a final volume of 100 mL (RPMI 1640 + 10% FCS). The purity of the sorted cells was routinely 85%. Plates were incubated at 37°C and 5% CO_2_ for 3 h, and NK cell movement toward the outer well was quantified by counting under a light microscope.

### Cytotoxicity assay

The cell cytotoxicity assay was performed as described previously [18]. Briefly, mouse hepatoma cells (Hepa-1c1c7), purchased from the ATCC (Manassas, VA, USA), were used as target cells, labeled with Na_2_[^51^Cr]O_4_, and then incubated with effector cells in round-bottomed 96-well plates for 4 h. Cytotoxicity, as indicated by ^51^Cr release, was calculated as a percentage according to the following equation: cytotoxicity (%) = [(cpm of experimental release − cpm of spontaneous release)]/[(cpm of maximum release − cpm of spontaneous release)] × 100. All assays were performed in triplicate.

### Statistical analysis

Student’s *t*-tests were performed for pairwise comparisons of parameters. Paired student’s *t*-tests were performed to compare differences within the same individual. Statistical differences in cytotoxicity were analyzed by ANOVA. A *p*-value of less than 0.05 was considered to indicate statistical significance. Values are expressed as means ± standard error of the mean. IBM SPSS Statistics for Windows, version 23.0 (SPSS Japan Inc., Tokyo, Japan) was used for calculations.

## Results

### TRAIL^+^ NK cells strongly express CXCR3

**Fig 1:**
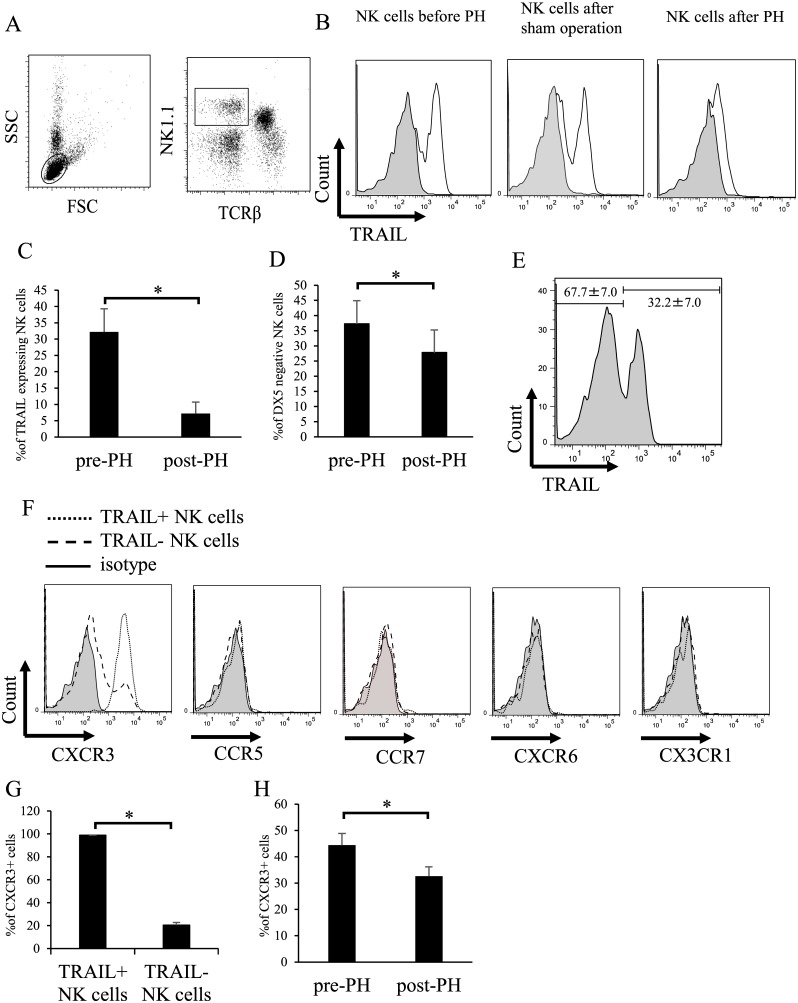
TRAIL^+^ hepatic NK cells express CXCR3. Liver mononuclear cells were isolated from untreated or partially hepatectomized B6 mice, and the expression of TRAIL, DX5, or various chemokine receptors was determined. (A) Liver mononuclear cells were stained with anti-NK1.1, anti-TCRβ, and propidium iodide. NK1.1^+^TCRβ^−^ NK cells were then gated for the analysis of other markers. (B) Representative histograms of TRAIL on NK1.1^+^TCRβ^−^ liver NK cells in mice before partial hepatectomy, after sham operation, or after partial hepatectomy are shown. (C) The proportions of TRAIL^+^ cells among NK1.1^+^TCRβ^−^ liver NK cells are shown before and after partial hepatectomy (n = 10). (D) Liver-resident (DX5^−^) NK cell populations decreased significantly after partial hepatectomy (n = 7). (E) Two liver NK cell populations were distinguished by TRAIL expression (n = 10). (F) TRAIL^+^ liver NK cells expressed only CXCR3. (G) The expression of CXCR3 was higher in TRAIL^+^ liver NK cells compared to that in TRAIL^−^ liver NK cells (n = 5). (H) CXCR3^+^ liver NK cell population decreased significantly after partial hepatectomy (n = 5). Data are expressed as means ± SD. Statistical differences were detected by paired *t*-tests (*p < 0.05).

Following partial hepatectomy in C57BL/6 mice, we observed that the number of TRAIL^+^ NK cells decreased significantly in the liver (Fig 1A, 1B and 1C). Liver-resident DX5^−^ NK cell numbers also decreased in the liver after partial hepatectomy (Fig 1D). As expected, the number of TRAIL^+^ NK cells did not change after sham operation (Fig 1B). As NK cells express several chemokine receptors with great heterogeneity [22, 23], we hypothesized that chemokine signaling is involved in TRAIL^+^ NK cell trafficking in the liver. To test this, we comprehensively analyzed the expression of chemokine receptors on intrahepatic NK cells by flow cytometry. Notably, almost all TRAIL^+^ NK cells exhibited high expression of CXCR3, whereas only a limited subpopulation of TRAIL^−^ NK cells expressed CXCR3 in the liver (Fig 1E, 1F and 1G). In parallel to TRAIL expression, CXCR3 expression on liver NK cells decreased significantly after hepatectomy (Fig 1H)

### CXCL9 levels decrease substantially in hepatic tissue after partial hepatectomy

**Fig 2:**
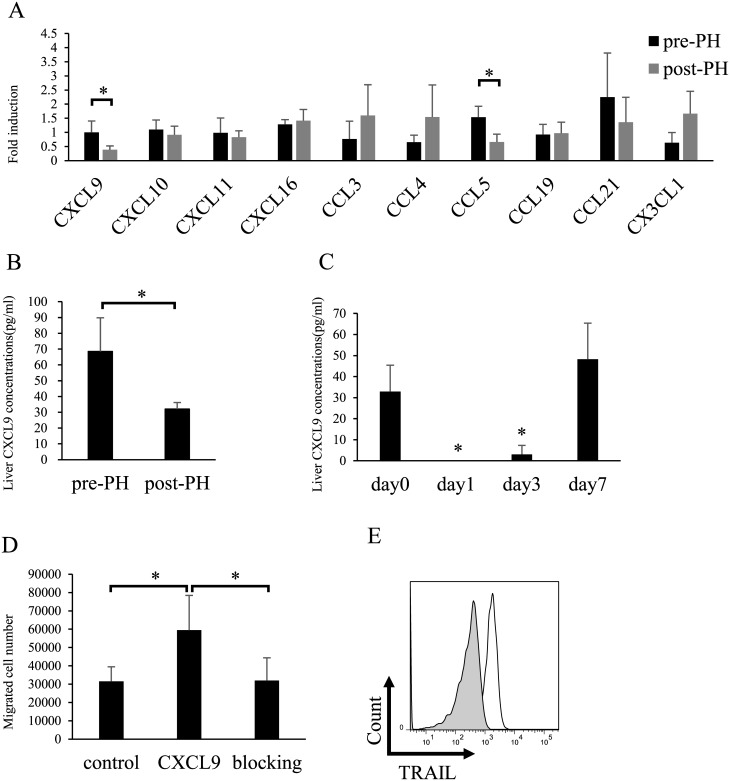
Intrahepatic CXCL9 expression decreases significantly after hepatectomy. (A) Gene expression levels of various chemokines in the liver were analyzed by quantitative real-time PCR. Numbers represent gene expression levels of various chemokines before and 3 d after partial hepatectomy (n = 6). (B) Protein levels of hepatic CXCL9 were measured by cytometric bead arrays before and after partial hepatectomy. The numbers represent the liver concentrations of CXCL9 for each group (n = 5). (C) Kinetics of liver CXCL9 after partial hepatectomy, as measured by cytometric bead array (n = 3). (D) Migration of liver NK cells in the presence of CXCL9 compared with that in presence of an isotype control. A CXCL9-blocking antibody attenuated migration (n = 5). (E) Representative histogram of TRAIL expression on migrating NK cells into the outer well. Shaded and open curves represent isotype-matched controls and TRAIL staining, respectively. Data are expressed as means ± SD. Statistical differences were detected by paired Student’s *t*-tests (*p < 0.05).

Next, we analyzed changes in intrahepatic chemokines before and 3 d after hepatectomy. The levels of various chemokines were measured in the liver by quantitative real-time PCR. The mRNA expression of *CXCL9*, which is a specific ligand of CXCR3, decreased significantly in the liver after partial hepatectomy (*p < 0.05; Fig 2A). CBA results revealed that protein levels of CXCL9 decreased significantly after partial hepatectomy but did not change after sham operation (Fig 2B). Although the mRNA expression of *CCL5* decreased significantly in the liver after hepatectomy, NK cells in the liver did not express CCR5, which is a specific receptor of CCL5 (Figs 2A and 1E).

Focusing on the kinetics of CXCL9 in the liver tissue after hepatectomy, the concentration of this chemokine transiently decreased to undetectable levels, but recovered 7 d after hepatectomy (Fig 2C). Interestingly, the dynamic kinetics of CXCL9 resembled those of TRAIL expression on liver NK cells after hepatectomy, as demonstrated in our previous study [18].

Together with the exclusive expression of CXCR3 on TRAIL^+^ NK cells and altered levels of CXCL9 in the liver after hepatectomy, chemotaxis of liver-resident NK cells was suggested to be involved in phenotypic alterations in these cells. To test the chemotactic activity of CXCR3^+^ NK cells toward recombinant CXCL9, freshly isolated liver NK cells were plated in microchemotaxis chambers, and their migratory potential was evaluated. Liver NK cells showed enhanced chemotaxis toward CXCL9 *in vitro*, and most migratory NK cells expressed TRAIL (Fig 2D and 2E). As such, a CXCL9 blocking antibody attenuated the chemotaxis of liver NK cells (Fig 2D). Thus, TRAIL^+^ NK cells have the potential to migrate toward CXCL9.

### Liver-resident macrophages produce CXCL9 upon stimulation with IFN-γ

**Fig 3:**
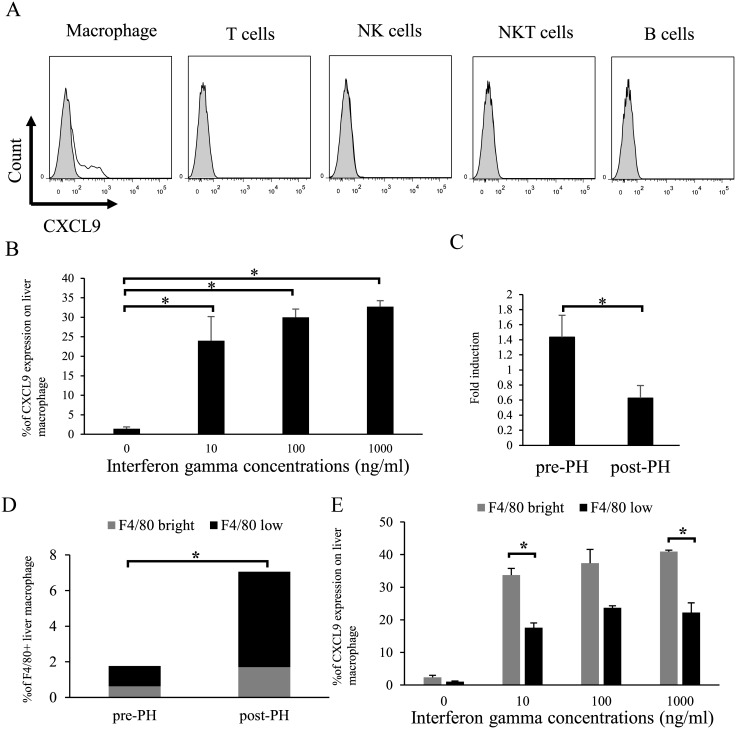
Liver-resident macrophages produce CXCL9 upon stimulation with IFN-γ in a dose-dependent manner. CXCL9 production was measured by intracellular staining using flow cytometry. Liver lymphocytes were cultured with various doses of IFN-γ for 4 h. (A) Representative histograms of CXCL9 production in liver lymphocytes. Shaded and open curves represent isotype-matched controls and anti-CXCL9 staining, respectively. (B) Liver macrophages, gated by anti-F4/80 staining, produced CXCL9 after stimulation with IFN-γ. Data are expressed as means ± SD (n = 2–3 mice per group). (C) IFN-γ expression was analyzed by qRT-PCR before and 3 d after 70% hepatectomy. Data are expressed as means ± SD (n = 6 mice). (D) Percentage of F4/80^+^ macrophages in liver mononuclear cells before and after hepatectomy (n = 4). Black bar, F4/80^low^ population. Gray bar, F4/80^bright^ population. (E) CXCL9 production by F4/80^bright^ and F4/80^low^ liver macrophages after stimulation with IFN-γ. Statistical differences were detected by Student’s *t*-tests (*p < 0.05).

CXCL9-dependent NK cell recruitment requires interferon (IFN)-γ [24–27]. To determine the population of liver cells that produces CXCL9, liver mononuclear cells were cultured with various doses of IFN-γ and subjected to intracellular cytokine staining for CXCL9. Among liver cells, macrophages predominantly produced high levels of CXCL9 upon stimulation with INF-γ, in a dose-dependent manner (Fig 3A and 3B). Consistent with the association between CXCL9 and IFN-γ levels in the liver, mRNA expression of *IFN-γ* in the liver decreased significantly after hepatectomy (Fig 3C). Regarding the number of liver macrophages, the proportion of macrophages defined as F4/80^+^ increased in the liver after hepatectomy (Fig 3D). Macrophages after hepatectomy consisted of a large population of F4/80^low^, which produced less CXCL9 than F4/80^bright^ macrophages (Fig 3D and 3E).

### Poly I:C induces CXCL9 production and preserves TRAIL^+^ NK cells in the liver after hepatectomy

**Fig 4:**
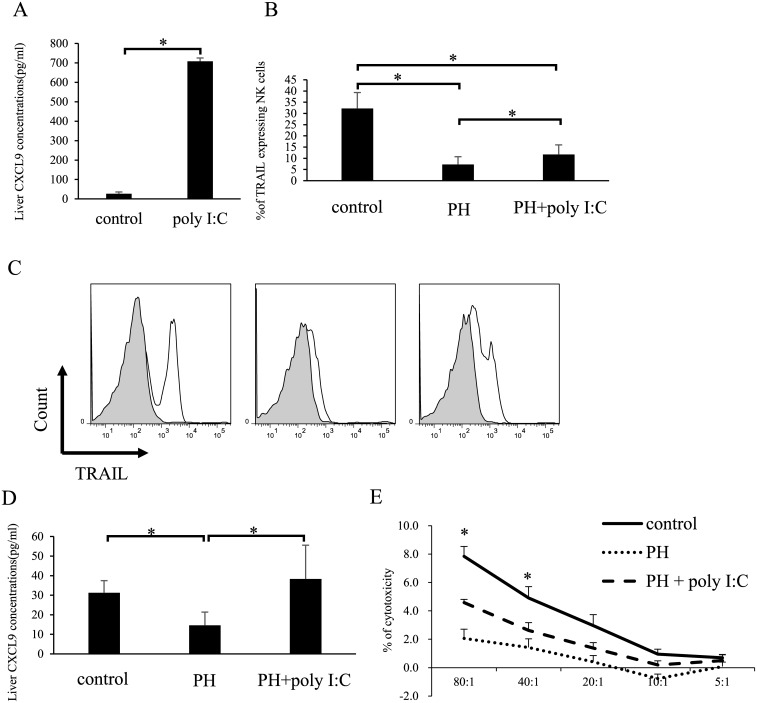
Poly I:C-mediated restoration of liver TRAIL^+^ NK cells depends on CXCL9. (A) Hepatic CXCL9 expression significantly increased after administration of poly I:C compared to that in untreated mice. CXCL9 concentration was measured 6 h after administration of poly I:C. Bar graphs represent the mean ± SD for each group (n = 6). (B) Poly I:C restored TRAIL^+^ NK cells in the liver 3 d after hepatectomy. B6 wild-type mice were injected with poly I:C 1 d after hepatectomy. TRAIL expression on NK cells is shown. (C) Representative histograms of TRAIL expression on liver NK cells. Shaded and open curves represent isotype-matched controls and TRAIL staining, respectively. (D) Poly I:C restored the CXCL9 concentration in the liver 3 d after hepatectomy. (E) Poly I:C restored the cytotoxicity of liver NK cells after hepatectomy. Data are expressed as means ± SD (n = 10 mice per group). Statistical differences were detected by Student’s *t*-tests (*p < 0.05).

**Fig 5:**
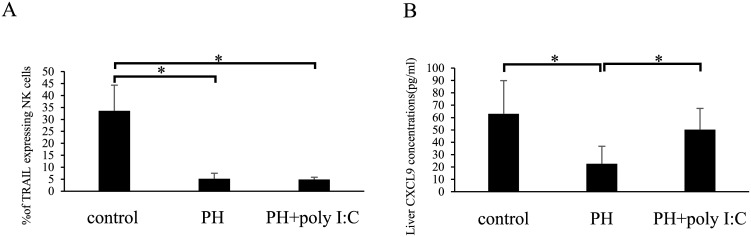
Poly I:C fails to restore hepatic TRAIL^+^ NK cells after hepatectomy in CXCR3 KO mice. (A) TRAIL^+^ NK cell numbers did not increase in hepatectomized mice after poly I:C treatment. CXCR3 KO mice were injected with or without poly I:C 1 d after hepatectomy and sacrificed on day 3. (B) Hepatic CXCL9 significantly increased after hepatectomy in CXCR3 KO mice. CXCR3 KO mice were injected with poly I:C 1 d after hepatectomy. Data are expressed as means *±* SD (n = 5 mice per group). Statistical differences were detected by Student’s *t*-tests (*p < 0.05).

According to a previous report, the IFN-γ-inducible CXCR3 ligands CXCL9 and CXCL10 play a role in NK cell recruitment to the inflamed liver [28]. We examined whether poly I:C, which induces IFN-γ via the TLR-dependent pathway [29], modulates hepatic CXCL9 levels after hepatectomy. Protein levels of CXCL9 were significantly upregulated in the liver after treatment with poly I:C (Fig 4A). This compound also significantly improved TRAIL expression on liver NK cells after hepatectomy compared with that in control hepatectomized mice without poly I:C treatment (Fig 4B and 4C). In addition, poly I:C restored hepatic CXCL9 concentrations and the cytotoxicity of liver NK cells, even after hepatectomy (Fig 4D and 4E). Poly I:C treatment partially restored TRAIL expression on liver NK cells from wild-type (WT) mice (Fig 4B and 4C), but failed to improve TRAIL expression on hepatic NK cells from *CXCR3*^*-/-*^ mice (Fig 5A). As expected, poly I:C increased hepatic CXCL9 concentrations in both *CXCR3*^*-/-*^ and WT mice (Fig 5B). Thus, poly I:C induced CXCL9 production and preserved TRAIL^+^ NK cells in the liver, even after hepatectomy.

## Discussion

We found that TRAIL^+^ NK cells exclusively express CXCR3, and not other chemokine receptors, in the livers of naïve mice, indicating that its corresponding ligands CXCL9, CXCL10, and/or CXCL11 might participate in the maintenance of liver-resident TRAIL^+^ NK cells. Partial hepatectomy resulted in a remarkable reduction in CXCL9 levels, but did not influence CXCL10 or CXCL11 expression in liver tissues, suggesting that the CXCL9–CXCR3 axis plays a pivotal role in the liver-specific distribution of TRAIL^+^ NK cells in mice.

A tissue-resident population of NK cells in the liver has recently been described to have the distinctive ability to elicit tumoricidal effects [18, 30]. Since NK cells express unique repertoires of chemokine receptors and adhesion molecules that are critical for tissue-specific trafficking, factors regulating the maintenance of these liver-resident NK cells likely involve their distinct chemotaxis to the liver [31]. The role of chemotaxis in the liver-restricted distribution of NK subsets might resemble the tumor-preferential distribution of NK subsets. The chemokine expression pattern in tissues is modified after neoplastic transformation, influencing the relative proportion of NK subsets that infiltrate the tissues [26]. In mice, it has also been shown that NK cells, which are normally excluded from the lymph nodes, are rapidly recruited to lymph nodes in a CXCR3-dependent manner upon the induction of T helper cell type 1 responses [32]. In addition, exogenous application of the CXCR3 ligand CXCL10 to tumor tissues, or ectopic expression of CXCL10 by tumor cells, has been demonstrated to increase the number of tumor-infiltrating NK cells expressing CXCR3 and to prolong NK cell-dependent survival in mice [27]. Despite the current knowledge on NK cell chemotaxis toward tumors, details regarding the CXC chemokine network that attracts NK cells to the liver remain to be elucidated.

To investigate the significance of CXCR3 on TRAIL^+^ NK cells, we analyzed the phenotypes of liver-resident NK cells in *CXCR3*^*-/-*^ mice in a preliminary experiment. Despite the expectation that a lack of CXCR3 on TRAIL^+^ NK cells would prevent the distribution of those cells in the liver of *CXCR3*^*-/-*^ mice (in which liver CXCL9 levels are similar to those in WT mice), the proportion of TRAIL^+^ NK cells among liver-resident lymphocytes in *CXCR3*^*-/-*^ mice did not differ from that in WT mice (S1 Fig). This might be explained by the previous demonstration that precursors of TRAIL^+^ DX5^−^ NK cells develop from a distinct lineage of bone marrow hematopoietic cells that arises in the fetal liver and persists in the livers of adult mice [33]. Even in the absence of the CXCL9–CXCR3 axis, the fact that the *CXCR3*^*-/-*^ genotype did not influence the proportion of TRAIL^+^ NK cells in mice might be attributed to the sequential development of TRAIL^+^ NK cells in the liver. Of note, partial hepatectomy decreased the proportion of hepatic TRAIL^+^ NK cells in *CXCR3*^*-/-*^ mice similar to that observed in WT mice. These results suggest that unknown receptors for CXCL9 are expressed on TRAIL^+^ NK cells and could be involved in their liver-specific distribution. Consistent with this assumption, poly I:C-induced CXCL9 supplementation in the liver significantly restored liver-resident TRAIL^+^ NK cells in WT mice, but not in *CXCR3*^*-/-*^ mice, after partial hepatectomy, indicating the dominant, but not exclusive role of CXCR3 in the CXCL9-dependent attraction of liver-resident NK cells.

We confirmed that liver macrophages produce CXCL9 in response to IFN-γ in a dose-dependent manner *in vitro*. This result is consistent with that of a previous study showing that peritoneal macrophages produce CXCL9 after stimulation with IFN-γ [34]. As liver cells are exposed to a variety of exogenous antigens via the portal vein [35], various liver-resident hematopoietic cells are consequently induced to release anti-inflammatory mediators, including IFN-γ, even during steady-state conditions [36]. Another possible explanation for the association between immune status and hepatectomy is that liver NK cells express the T cell Ig and ITIM domain (TIGIT), which acts as a negative regulator. Hepatectomy increases levels of TIGIT on liver NK cells, which could cause IFN-γ levels to decrease in the liver [37]. Regarding the liver macrophages, the number of F4/80^+^ macrophages unexpectedly increased in the liver after partial hepatectomy. Interestingly, the intensity of F4/80 on liver macrophages significantly decreased after hepatectomy. Schulz et al. [38] reported that liver macrophages exhibited two types of development, including F4/80^bright^ and F4/80^low^ macrophages. F4/80^low^ liver macrophages are derived from bone marrow, while F4/80^bright^ liver resident macrophages develop in the liver. Although the function of F4/80^low^ liver macrophages remains unclear, the increased proportion of liver macrophages after hepatectomy may reflect the movement of macrophages from the bone marrow to the liver, as liver macrophages in this study consisted of a large population of F4/80^low^ cells after hepatectomy. F4/80^low^ liver macrophages produce less CXCL9 upon IFN-γ stimulation than F4/80^bright^ liver resident macrophages. We also found that *IFN-γ* mRNA in the mouse liver, which was detectable under naïve conditions, significantly decreased after hepatectomy. Such dramatic alterations in IFN-γ levels likely mediate the reduction in CXCL9 levels after hepatectomy, despite the increased number of F4/80^+^ macrophages.

We used poly I:C to increase the hepatic concentration of CXCL9; however, poly I:C is not available for clinical use owing to its toxic side effects, including shock, renal failure, coagulopathies, and hypersensitivity reactions [39]. Some researchers have developed chimeric TLR3 agonists as replacements for poly I:C; however, these are not yet clinically available [40]. If these reagents maintain high levels of CXCL9 in the liver, such treatments could prevent impaired host defense after hepatectomy. As such, we attempted to inject recombinant CXCL9 by various methods, including portal injection and continuous peritoneal injection; however, TRAIL^+^ NK cells were not maintained in the liver after hepatectomy. A possible reason for this is the very short half-life of recombinant CXCL9 (24 min) [41]. Exploring these clinical treatments represents a future challenge for pharmaceutical scientists.

In addition to the modulation of chemotaxis pathways, the decrease in TRAIL^+^ NK cells after hepatectomy might be explained by the inhibitory effects of local microenvironments of the liver on intrahepatic differentiation into TRAIL^+^ DX5^−^ NK cells from precursors that persist in the livers of mice [33]. It has been reported that mRNA encoding IL-15, which is essential for NK cell maturation and survival, increases after partial hepatectomy [42]. Since the expression of Tbx21, which is required for differentiation into TRAIL^+^ NK cells [19], is significantly decreased in the presence of IL-15 [43], increased IL-15 levels after hepatectomy might interfere with the hepatic development of TRAIL^+^ NK cells, but not TRAIL^−^ NK cells. This possibility requires future experiments for confirmation.

## Conclusions

We found that the expression of intrahepatic CXCL9, which was predominantly secreted by liver macrophages in parallel with IFN-γ, is correlated with the dynamics of TRAIL^+^ NK cells expressing CXCR3 in the liver after partial hepatectomy. These TRAIL^+^ NK cells were chemotactically attracted by CXCL9, whose expression was transiently, but significantly decreased by partial hepatectomy. Overall, these findings indicated that partial hepatectomy remarkably reduced the proportion of TRAIL^+^ NK cells in the liver by inhibiting the CXCL9–CXCR3 axis in mice.

## Supporting information

S1 Fig TRAIL expression on liver NK cells. Liver mononuclear cells were isolated and stained for NK1.1, TCRβ, and TRAIL. Representative histogram showing TRAIL expression on TCRβ^−^NK1.1^+^ liver NK cells in wild-type B6 mice and CXCR3 KO mice. Shaded and open curves represent isotype-matched controls and TRAIL staining, respectively.
(EPS)

S1 Table Information of primers related with quantitative RT-PCR and sequencing analysis. (XLSX)
